# Empirical 5/3 Spectral Scaling in Contextual Representations of Language

**DOI:** 10.3390/e28101063

**Published:** 2026-09-27

**Authors:** Zhongxin Yang, Chun Bao, Yuanwei Bin, Xiang I. A. Yang, Yipeng Shi, Shiyi Chen

**Affiliations:** 1 College of Engineering, Peking University, Beijing 100871, China; zhongxinyang@stu.pku.edu.cn (Z.Y.); 1800011035@pku.edu.cn (C.B.); ypshi@coe.pku.edu.cn (Y.S.);; 2 Ningbo Institute of Digital Twin, Eastern Institute of Technology, Ningbo 315200, China; 3 Shenzhen Tenfong Technology Co., Ltd., Shenzhen 518000, China; 4 Department of Mechanical Engineering, Pennsylvania State University, State College, PA 16802, USA

**Keywords:** contextual embeddings, spectral scaling, semantic organization

## Abstract

Natural language is a complex system that exhibits robust statistical regularities. Here, we represent text as a trajectory in a high-dimensional embedding space generated by transformer-based language models, and quantify scale-dependent fluctuations along the token sequence using an embedding-step signal. Across multiple languages and corpora, the resulting power spectrum exhibits a robust power law with an exponent close to 5/3 over an extended frequency range. This scaling is observed consistently in contextual embeddings from both human-written and AI-generated text, but is absent in static word embeddings and is disrupted by randomization of token representation order. These results show that the observed scaling reflects multiscale, context-dependent organization rather than lexical statistics alone. These results identify an empirical spectral regularity in contextual language representations and provide a quantitative, model-agnostic benchmark for studying their multiscale structure.

## 1. Introduction

Natural language is a highly complex system. Its hierarchical structure, which balances strict grammatical constraints with immense expressive variability, manifests in robust statistical regularities across languages and domains. Classic empirical laws, such as Zipf’s law for heavy-tailed word frequencies [1], provide macroscopic constraints on linguistic organization. Furthermore, recent work on linguistic “criticality” suggests that text may balance order and randomness in a regime that supports rich variability [2,3]. Long-range correlations in texts have also been linked to hierarchical linguistic and semantic organization [4]. Together, these findings motivate the view of language as a complex system with structure spanning multiple scales. However, most existing laws are formulated in terms of observable text statistics, and it remains unclear whether representation-level signatures—especially those learned by modern neural language models—exhibit similarly simple and reproducible multiscale behavior.

Transformer-based language models [5] provide a natural setting to address this question. Models such as BERT [6] and GPT-style transformers [7] map a token sequence into a sequence of high-dimensional vectors (contextual token representations) whose values depend on surrounding words. This contextual dependence distinguishes transformers from static word embeddings such as Word2Vec [8] and GloVe [9]. Contextual embeddings thus offer a model-defined representation of language-in-context, enabling quantitative analysis beyond token counts alone.

Spectral analysis provides a compact description of multiscale organization in complex systems, and power laws can serve as empirical fingerprints. Here, we use spectral scaling as a model-agnostic tool to characterize multiscale structure in contextual language representations, without assuming a specific physical mechanism.

In this paper, we analyze texts as sequences of contextual token representations and quantify scale-dependent structure along token position. To emphasize local variation while reducing sensitivity to slow document-level drift, we study token-to-token embedding differences (the embedding-step signal) rather than the raw embedding trajectory. Across multiple languages and diverse corpora—including both human-written and AI-generated text—the power spectrum of embedding-step changes follows a robust power law with an exponent close to 5/3 over an extended frequency range. This scaling is consistently observed for transformer-based contextual embeddings, but is absent in static embeddings and is eliminated by randomizing token-representation order, indicating that it arises from context-dependent organization rather than lexical statistics alone. These results identify a reproducible spectral fingerprint of contextual language embeddings and provide a quantitative benchmark for comparing multiscale structure across languages, corpora, and model families.

## 2. Materials and Methods

We constructed corpora spanning four languages (Chinese, English, German, and Japanese) and two sources (human-authored and AI-generated). Chinese primarily uses a logographic writing system and does not rely on whitespace-delimited word boundaries. Japanese uses a mixed kanji–kana writing system, also lacks whitespace-delimited word boundaries, and has agglutinative morphology. English and German use alphabetic scripts and whitespace-delimited orthography, where German has richer inflectional morphology and productive compounding than English. The multilingual comparison is intended to test the reproducibility of the observed scaling across four contrasting language–model–tokenizer settings. Extending the analysis to a broader typological sample is a valuable direction for future work. For each language, tokenization followed the native tokenizer of the corresponding embedding model, and all samples were truncated to a fixed length of L=1200 tokens to provide the same nominal number of representation steps for spectral analysis. Samples shorter than 1200 tokens were excluded. Human-authored corpora cover multiple domains. AI-generated corpora were produced using the DeepSeek-V3 model [10]. Representative topic lists and prompt templates are provided in Appendix A. The domain coverage was not identical across languages: Chinese and English include scientific writing, news reports, and movie reviews, whereas German and Japanese are restricted to news corpora because of the availability of suitable long-form datasets.

We embedded each token sequence using both contextual and static embedding models. Contextual representations were obtained from transformer-based models, including language-specific BERT variants for Chinese, German, and Japanese, and an open-source GPT-style model [11] for English. Static embeddings were obtained from Word2Vec (Chinese) and GloVe (English). All models were accessed through Hugging Face or official repositories [12,13].

Let $x\left(t\right)\in R^{d}$ denote the contextual token embedding at token position *t*, where *d* is the embedding dimension. Unless otherwise stated, x(t) was taken from the final hidden layer of the contextual model. The final hidden layer was used for the main analysis because it is the standard output representation after processing by all transformer blocks and therefore provides the most fully contextualized representation available within each model.

To quantify scale-dependent structure along token positions while reducing sensitivity to slow document-level drift, we analyzed the token-to-token embedding difference signal:

(1) v(t)=x(t+1)−x(t),

which we interpret as informational fluctuation of the semantic trajectory. For each sample and each embedding dimension, we computed the discrete Fourier transform of the embedding-step sequence along token positions:

$$u_{j}\left(f_{k}\right)=\sum_{t=0}^{N-1}v_{j}\left(t\right)\exp\left(-if_{k}t\right),\;f_{k}=\frac{2\pi k}{N},\;k=0,\ldots ,\left\lfloor \frac{N}{2}\right\rfloor ,$$

where *N* is the number of embedding steps and j=1,…,d indexes the embedding dimension. Thus, *f* denotes the discrete angular frequency conjugate to token position, with units of radians per token. We denote the highest positive resolved frequency by $f_{\max}=f_{\left\lfloor N/2\right\rfloor }$ and display spectra as functions of the dimensionless normalized frequency $f/f_{\max}$.

This first-difference operation has a simple spectral consequence. Let $\hat{x}\left(f\right)$ denote the discrete Fourier transforms of x(t). Then,

$$u\left(f\right)=\left(e^{if}-1\right)\hat{x}\left(f\right),$$

so that the power spectral density has the following relation:

$$E_{u}\left(f\right)=4\sin^{2}\left(f/2\right)E_{x}\left(f\right).$$

At low normalized frequencies, $4\sin^{2}\left(f/2\right)\approx f^{2}$, implying that first differencing acts as a high-pass filter and shifts the local spectral exponent by approximately two.

The power spectral density describes the scale-resolved variance of the embedding-step signal and is not interpreted as a physical energy spectrum. The one-dimensional power spectral density was then formed by taking the ensemble average across the *d* spatial dimensions and over the corpus samples. To allow universal comparison across different models and dimensionalities, the dimension-averaged spectrum $\bar{E}\left(f\right)$ was normalized by the total variance, $\sigma^{2}=\int \bar{E}\left(f\right)\;d\left(f/f_{\max}\right)$.

## 3. Results

Figure 1a shows the dimension-averaged spectrum

(2) $$\bar{E}\left(f\right)=\frac{1}{d}\sum_{j=1}^{d}E_{j}\left(f\right),$$

for contextual embedding in four language/model configurations. For each document, we compute $\bar{E}\left(f\right)$ and then average the resulting spectra across all documents within the same configuration. Across all configurations, the spectra exhibit a clear power-law regime over an extended frequency range, and the human and AI curves closely collapse in each panel (Figure 1a). Here, we estimate the scaling exponent by an ordinary least-squares fit on log–log axes over the frequency window indicated by the two vertical lines (approximately $f/f_{\max}\in \left[2\times 10^{-1},\;2\times 10^{-2}\right]$), which spans about one decade. Since *f* is the discrete angular frequency conjugate to token position and $f_{\max}=\pi$ radians per token, the characteristic period associated with a frequency *f* is $\lambda \;=\;2\pi /f\;=\;2/(f/f_{\max})$ token positions. Thus, the fitting interval $f/f_{\max}\in \left[2\times 10^{-2},2\times 10^{-1}\right]$ corresponds approximately to periods of 10–100 token positions. Goodness of fit was assessed using the coefficient of determination $R^{2}$ computed over the same fitting window. Across all tested language–source configurations, the dimension-averaged spectra yielded $R^{2}>0.99$, indicating that the power-law model provides an excellent description of the spectra within the selected scaling range. The fitted exponent is consistently close to five-thirds. The robustness of this result to different analyzed sequence lengths is further examined in Appendix B.

The collapse of the averaged spectra raises a natural question: is the observed scaling confined to a small subset of embedding dimensions, or is it broadly expressed across the representation space? To assess this, we compute the per-dimension spectrum $E_{j}\left(f\right)$ for each component j=1,…,d and visualize the normalized spectra as polar heatmaps (Figure 1b), with frequency on the radial axis and embedding-dimension index on the angular axis. Across languages and architectures, the heatmaps reveal similar spectral structure over a large fraction of dimensions, indicating that the scaling is not driven by a small number of dominant axes. Consistent with this visual evidence, the average fraction of embedding dimensions whose fitted exponents fall within ±10% of 5/3 exceeds 90% across the tested language–source configurations.

We next test whether the scaling is specific to contextual processing and ordered token-representation sequences using two contrastive analyses. First, we replace contextual embeddings with static word embeddings (Word2Vec and GloVe), which do not incorporate surrounding context. Second, we destroy contextual structure by randomly shuffling the token-representation order within each sequence prior to spectral analysis. Figure 2a summarizes the fitted scaling exponents across all configurations. Contextual embeddings cluster near five-thirds for all four languages and for both human-written and AI-generated text. In contrast, static embeddings do not exhibit the same scaling behavior, and token-representation-order randomization eliminates the five-thirds regime, producing substantially different exponents. Together, these controls indicate that the observed scaling is not a generic property of embedding vectors alone, but instead reflects context-dependent organization that depends on sequential order.

Finally, to examine how the fitted spectral exponent depends on representation depth, we repeat the analysis using embeddings extracted from intermediate transformer layers. Figure 2b plots the fitted exponent versus layer index for human-authored text. For the BERT configurations (13 layers in the present setup), the exponent generally moves toward 5/3 with increasing depth: DE and JP approach 5/3 in an almost monotonic manner, with a relatively flat plateau near α≈1.85 around layers 6–9, whereas CN exhibits a mid-layer dip to α≈1.5 around layer 7, followed by larger fitted exponents in the later layers. For the GPT configuration (25 layers), the fitted exponent decreases to α≈1.5 by approximately layer 7, remains near this level until about layer 19, and increases around layer 21. The deepest GPT layers yield fitted exponents close to 5/3. These results indicate that the spectral exponent is layer-dependent, while the deepest contextual layers tend to provide the most consistent estimates near 5/3 in the tested models.

## 4. Discussion and Conclusions

We report a robust five-thirds power-law scaling in the spectrum of token-to-token embedding differences produced by contextual language models. The scaling is observed across four languages and diverse corpora, including both human-written and AI-generated text, and appears consistently in transformer-based architectures (BERT and GPT-style models). In contrast, the scaling is absent in static word embeddings (Word2Vec and GloVe) and disappears when token-representation order is randomized, indicating that it depends on contextual representations and sequential structure. Together, these results identify a reproducible spectral signature within the tested final-layer contextual language-model settings.

The numerical occurrence of an exponent with magnitude close to 5/3 motivates comparison with the Kolmogorov spectrum of high-Reynolds-number turbulence [14]. This comparison is intended only as an interpretive reference. In three-dimensional turbulence, the $k^{-5/3}$ energy spectrum is associated with the Navier–Stokes equations, the inertial range, and a scale-to-scale energy flux. None of these ingredients is established by the present analysis of token-to-token embedding differences. In particular, we neither derive the observed power law from a governing equation nor measure a transfer of energy, information, or semantic content across token scales. Whether an information-theoretic mechanism underlies this representation-level spectral regularity therefore remains an open question.

Within the present scope, the fitted exponent is useful as a descriptive statistic for comparing contextual representations. Its recurrence across the tested language–corpus–model configurations, together with the contrasting results for static embeddings and shuffled token-representation sequences, suggests that it can provide a compact summary of multiscale structure in the tested representations. It may therefore complement downstream benchmarks in systematic comparisons across corpora, languages, and model families.

## Figures and Tables

**Figure 1:**
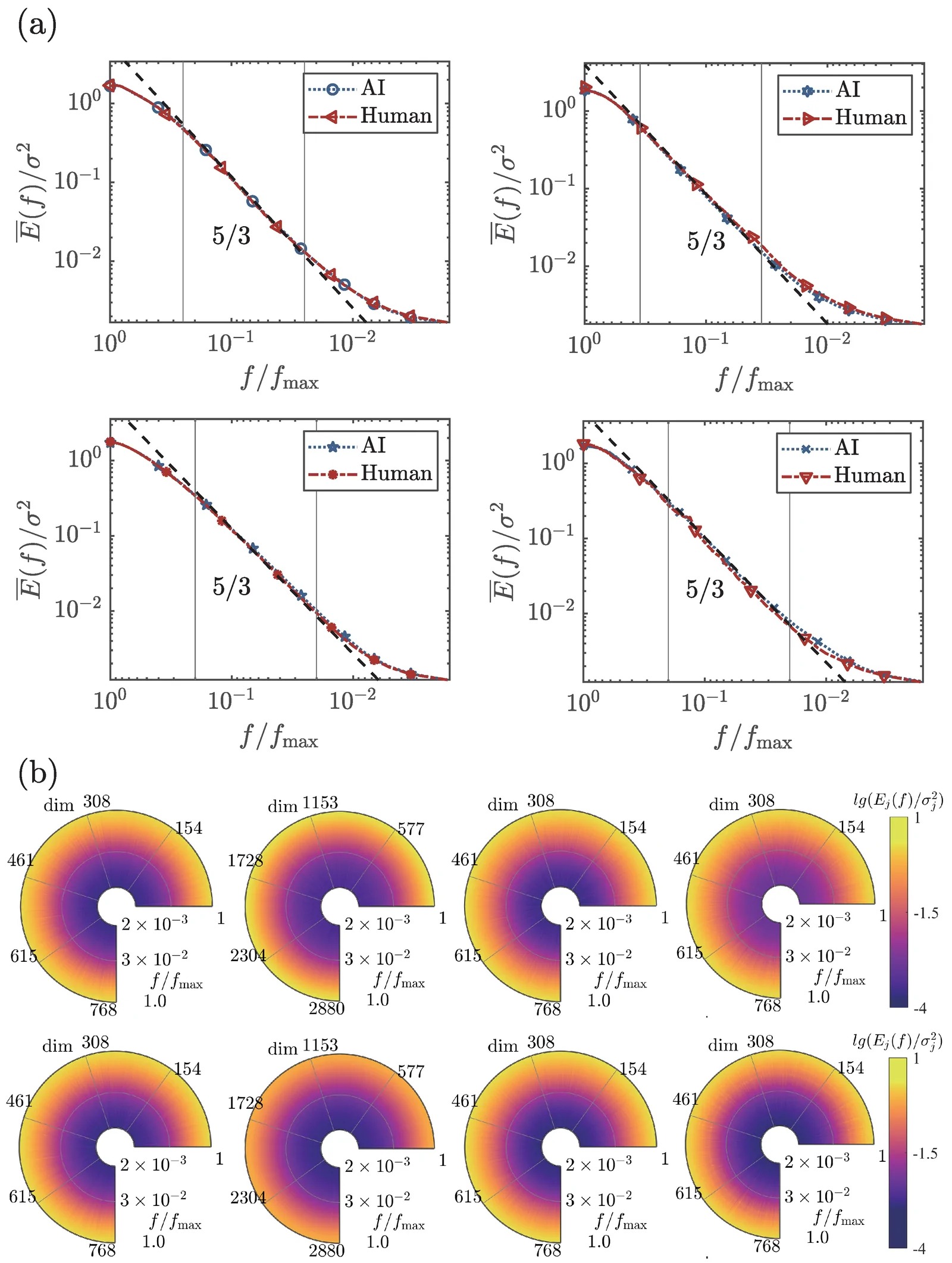
(**a**) Power spectra of contextual embedding-step sequences. The spectra are normalized by the variance $\sigma^{2}=\int_{0}^{1}\bar{E}\left(f\right)\;d\left(f/f_{\max}\right)$. The dashed line indicates the +5/3 slope. The four panels are Chinese/BERT (top left); English/GPT-OSS (top right); German/BERT (bottom left); Japanese/BERT (bottom right). (**b**) Polar heatmaps of normalized per-dimension power spectra. The radial axis represents frequency (log scale), and the angular axis represents embedding dimension index. Top row: AI; bottom row: human; from right to the left: Chinese, English, German, Japanese.

**Figure 2:**
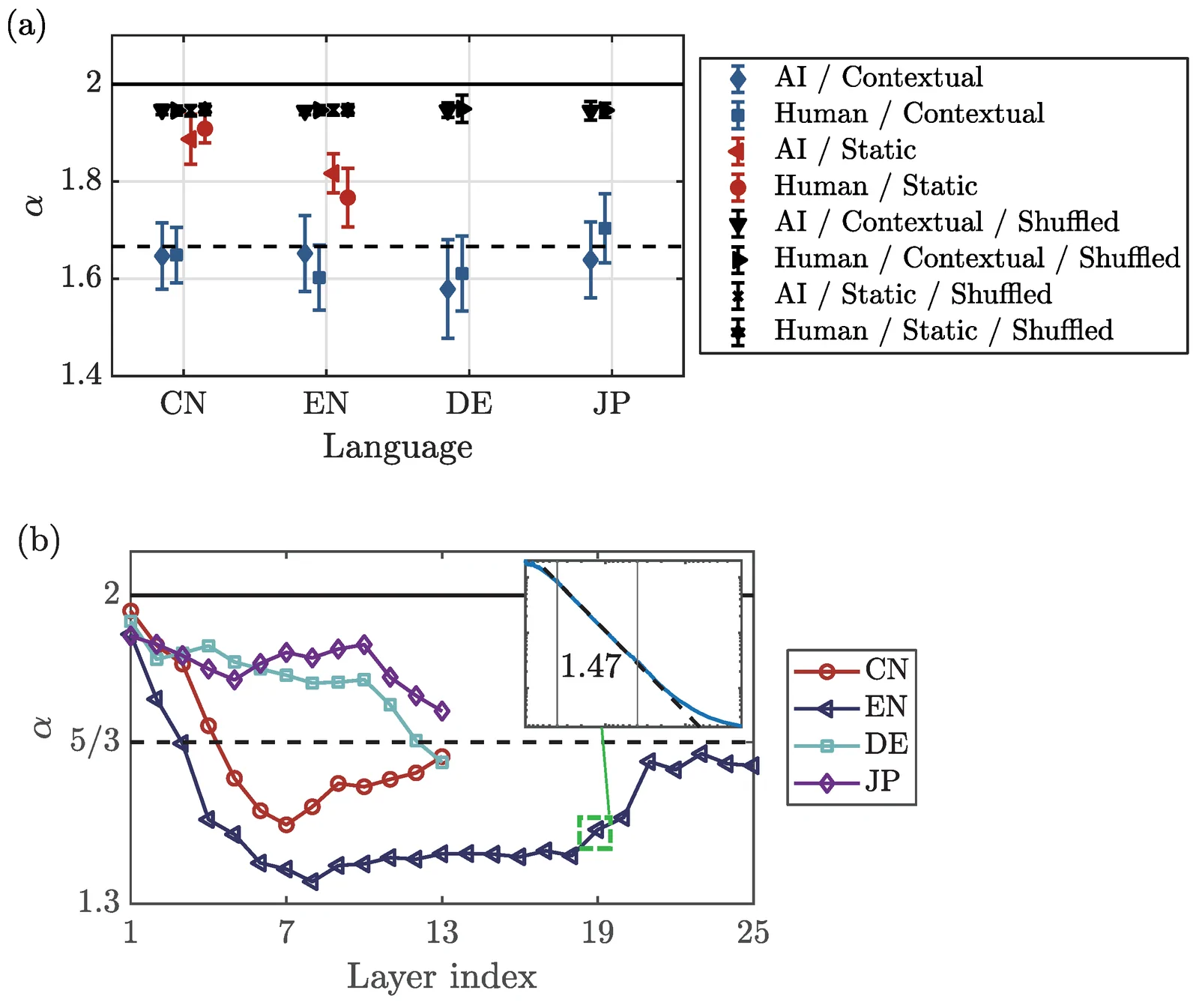
(**a**) Summary of fitted scaling exponents α. ‘CN’, ‘EN’, ‘DE’, and ‘JP’ denote Chinese, English, German, and Japanese, respectively. Error bars show variability across embedding dimensionalities (one standard deviation). (**b**) Fitted power-law exponent versus transformer layer index for human-authored text. The inset shows a representative mid-layer spectrum (highlighted by the green box) with a fitted power-law exponent α≈1.47.

**Figure A1:**
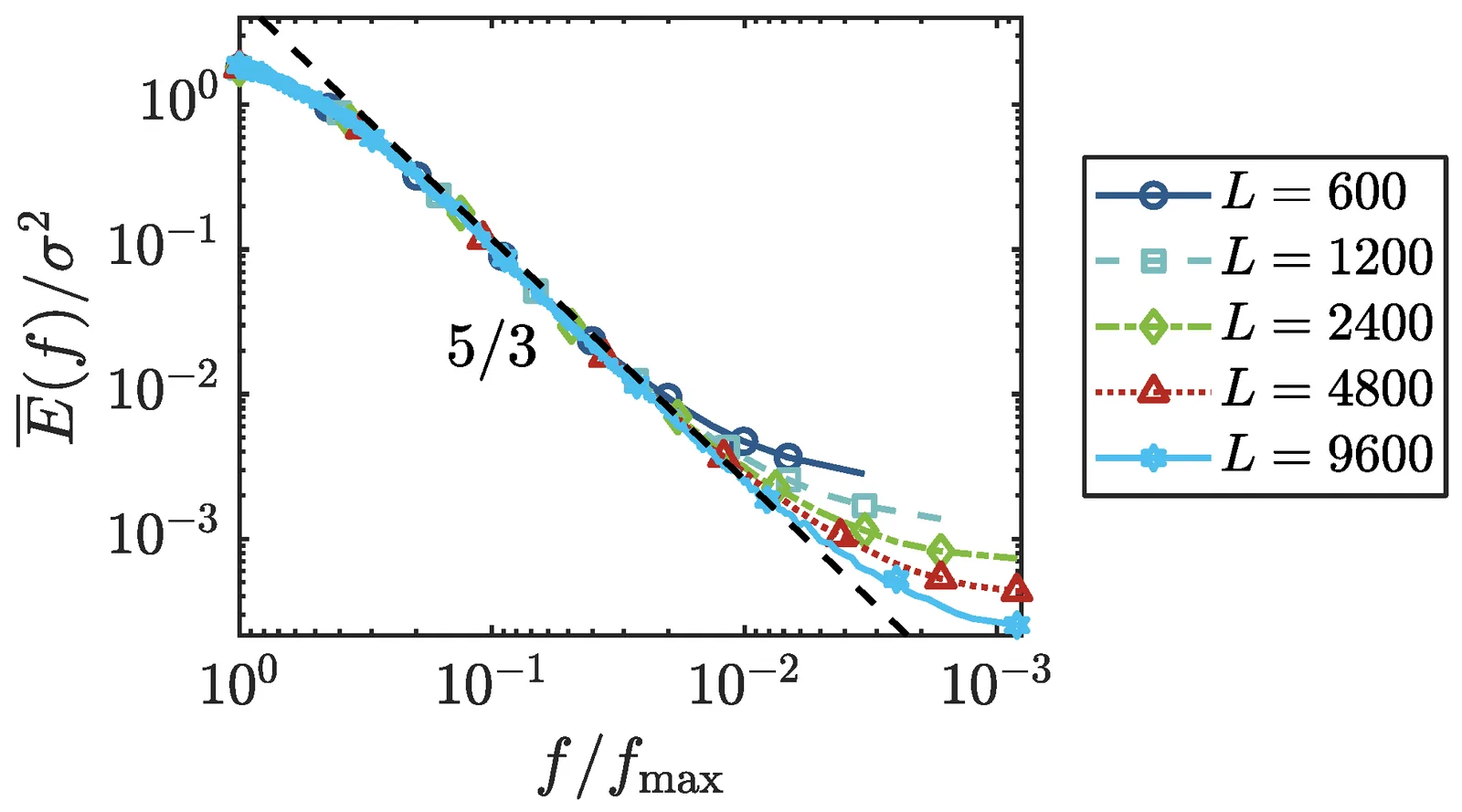
Dimension-averaged power spectra of embedding-step signals from human-authored Chinese scientific texts encoded with the Chinese BERT model. The analysis is repeated for token-sequence lengths L=600, 1200, 2400, 4800, and 9600. The dashed line indicates a slope with magnitude 5/3. With increasing *L*, the approximately power-law region extends toward lower normalized frequencies.

## Data Availability

Raw data are available from the author upon reasonable request.

## Appendix A. Corpus Details and Generation Methodology

We curated a multilingual corpus spanning Chinese, English, German, and Japanese to ensure the robustness of the observed spectral scaling across different linguistic structures. The text sequences were systematically collected from human-authored sources and correspondingly generated using artificial intelligence.

### Appendix A.1. Multilingual Corpus Composition

For Chinese and English, the dataset covers three primary domains: scientific/technical writing, news reports, and movie reviews; the Chinese corpus additionally includes Gaokao (college entrance exam)-style essays. The human-authored Chinese texts were drawn from CNKI research articles (primarily 2024–2025), CCTV news reports (2022–2025), and Douban movie reviews (e.g., *The Shawshank Redemption*). This subset comprises 1340 samples for BERT-based encoding and 814 for Word2Vec encoding. Corresponding AI-generated texts were produced for the same domains, totaling 1062 (BERT) and 761 (Word2Vec) samples.

Human-authored English texts include research papers from journals (e.g., Physical Review Letters/arXiv/Journal of Fluid Mechanics/Physics of Fluids), a cleaned CNN news corpus (2011–2022), and IMDB movie reviews. The resulting dataset contains 1228 samples for GPT encoding and 1052 for GloVe encoding. The AI-generated counterparts consist of 1465 (GPT) and 1263 (GloVe) samples.

The German and Japanese corpora are exclusively restricted to the news domain. Human German texts (N=428) were sourced from the 10kGNAD news dataset (2015–2016), and the corresponding AI-generated texts contain 428 samples. Human Japanese texts (N=500) are from a cleaned CC-News subset (2024), with 292 corresponding AI-generated samples.

### Appendix A.2. AI Text Generation Protocols

All AI corpora were generated using the DeepSeek-V3 model [10]. To ensure that the generated text provided sufficient length for multiscale spectral analysis, prompts were strictly controlled to produce long-form sequences: 600–800+ words per technical paper section, 800+ words per news report (enforcing standard journalistic structures), and over 1000 words for movie reviews.

Scientific Writing (Chinese and English): To cover a diverse semantic phase space, scientific texts were generated across 14 distinct research topics: Generative Artificial Intelligence, Quantum Cryptography, Brain–Computer Interfaces, Edge Computing, Graphene Batteries, 6G Network Architecture, Autonomous Driving Perception Algorithms, Digital Twin Cities, Bioprinting Technology, Zero-Knowledge Proofs, Controlled Nuclear Fusion, Starlink Communication, Exoskeleton Robotics, and Nanomedical Robots.

The prompt templates enforced strict academic standards, including minimum length, technical specificity, and logical rigor. The Chinese and English prompts were structured as follows, where placeholders like {topic} and {section_title} were dynamically injected:

你正在撰写一篇主题为《{topic}》的深度科技论文。当前请撰写章节：【{section_title}】。

要求： 1. 字数要求：本章节内容必须丰富翔实，字数不少于800字。2. 内容深度：包含具体的技术参数、理论推导或行业数据，拒绝空泛的套话。3. 风格：学术性强，逻辑严密。4. 仅输出本章节的正文内容，不需要重复标题。

You are writing a comprehensive academic paper on the topic "{topic}". Please write the section: [{section_title}].

Requirements: 1. Length: The content must be substantial. Please write at least 800 words for this section. 2. Depth: Include specific technical parameters, theoretical derivations, or industry data. Avoid vague generalizations. 3. Style: Professional academic English, rigorous logic. 4. Format: Output ONLY the body text of this section. Do not repeat the title.

Journalistic Reporting (Japanese and German): For the news domains, topics were selected to reflect contemporary macroscopic societal and economic themes. The Japanese topics included AI and Future Technology, Global Economy and the Japanese Market, Declining Birthrate and Aging Population, Climate Change, Medical Technology, International Politics, Globalization of the Anime/Manga Industry, Inbound Tourism, Renewable Energy, Educational Reform, Olympic Legacy, and Regional Revitalization. The prompt explicitly required the formal journalistic style 常体 typical of major Japanese publishers (e.g., Asahi, Yomiuri, Nikkei), structured with a lead paragraph, background analysis, and expert commentary:

テーマ「{category}」について、詳細なニュース記事を書いてください。要件：1. 具体的な出来事：この分野における架空のニュースイベント、または現在のトレンドを深く分析してください。2. タイトル：新聞記事らしい、プロフェッショナルで目を引く見出しをつけてください。3. 構成：リード文（5W1Hを含む）、本文（背景、詳細、データ分析）、専門家のコメント（架空の識者の発言を含む）、結び。4. 文体：「だ・である」調（常体）を使用し、日本の大手新聞社のスタイルで執筆してください。5. 長さ：本文は必ず800文字以上にしてください。

Similarly, the German news generation spanned analogous macroeconomic and societal topics: AI and Industry 4.0, European Markets, Energy Transition, Medical Research, EU Foreign Policy, E-Mobility, Digitalization, Modern Art, Sports Science, Sustainability, Smart Cities, and Food Security. The prompt mandated an objective, sophisticated tone characteristic of major German outlets (e.g., *FAZ, Süddeutsche Zeitung, Der Spiegel*):

Bitte schreiben Sie einen ausführlichen Nachrichtenbericht zum Thema {category}. Anforderungen: 1. Konkretes Ereignis: Erfinden Sie ein realistisches Szenario oder analysieren Sie einen aktuellen Trend. 2. Schlagzeile: Formulieren Sie eine professionelle und prägnante Überschrift. 3. Struktur: Enthalten Sie einen Datumszeile, einen starken Lead-Absatz (W-Fragen), Hauptteil mit Hintergrundinformationen und Analysen, Zitate von Experten (fiktiv aber plausibel) und ein Fazit. 4. Länge: Der Artikel muss mindestens 800 Wörter lang sein. Es soll sich um eine tiefergehende Reportage handeln. 5. Stil: Objektiv, seriös und anspruchsvoll.

## Appendix B. Sequence-Length Robustness Analysis

To examine the dependence of the observed scaling on the analyzed sequence length, we repeated the spectral analysis for human-authored Chinese scientific texts encoded with the Chinese BERT model. The analysis used token-sequence lengths of L=600, 1200, 2400, 4800, and 9600. For each value of *L*, the same preprocessing and spectral-estimation procedure as in the main analysis was applied.

Figure A1 shows that the spectra have comparable slopes over their common frequency range, with an exponent close to 5/3. As *L* increases, the approximately power-law portion of the spectrum extends toward lower normalized frequencies. This behavior is qualitatively analogous to the widening of a scaling interval with increasing scale separation in turbulence. However, in the present analysis *L* is a sequence length, not an analogue of the Reynolds number, and the observed interval is not identified as a physical inertial range. Because frequency is defined with respect to token position, the linguistic or information-theoretic mechanism underlying this length dependence remains an open question.
